# Cooking behaviors are related to household particulate matter exposure in children with asthma in the urban East Bay Area of Northern California

**DOI:** 10.1371/journal.pone.0197199

**Published:** 2018-06-06

**Authors:** Stephanie M. Holm, John Balmes, Dan Gillette, Kris Hartin, Edmund Seto, David Lindeman, Dianna Polanco, Edward Fong

**Affiliations:** 1 UCSF Benioff Children’s Hospital Oakland, Oakland, CA, United States of America; 2 University of California Berkeley, School of Public Health, Division of Epidemiology, Berkeley, CA, United States of America; 3 University of California San Francisco, Division of Occupational and Environmental Medicine, San Francisco, CA, United States of America; 4 University of California Berkeley, School of Public Health, Division of Environmental Health Sciences, Berkeley, CA, United States of America; 5 University of California Berkeley, Center for Information Technology Research in the Interest of Society, Berkeley, CA, United States of America; 6 University of Washington, Department of Environmental and Occupational Health Sciences, Seattle, WA, United States of America; 7 Department of Pediatrics, Kapiolani Medical Center for Women and Children, Honolulu, HI, United States of America; 8 University of Hawaii-Manoa John A. Burns School of Medicine, Honolulu, HI, United States of America; Cincinnati Children's Hospital Medical Center, UNITED STATES

## Abstract

**Background:**

Asthma is a common childhood disease that leads to many missed days of school and parents’ work. There are multiple environmental contributors to asthma symptoms and understanding the potential factors inside children’s homes is crucial.

**Methods:**

This is a dual cohort study measuring household particulate matter (PM_2.5_), behaviors, and factors that influence air quality and asthma symptoms in the urban homes of children (ages 6–10) with asthma; one cohort had cigarette smoke exposure in the home (n = 13) and the other did not (n = 22). Exposure data included measurements every 5 minutes for a month.

**Results:**

In the entire study population, a large contributor to elevations in indoor PM_2.5_ above 35 μg/m^3^ was not using the stove hood when cooking (8.5% higher, CI 3.1–13.9%, *p*<0.005). Median PM values during cooking times were 0.88 μg/m^3^ higher than those during non-cooking times (95% CI 0.33–1.42). Mean monthly household PM_2.5_ level was significantly related to the presence of a cigarette smoker in the home (10.1 μg/m^3^ higher, 95% CI 5.2–15.1, *p*<0.001) when controlling for use of the stove hood and proximity to major roadway. There was a trend toward increased odds of persistent asthma with increases in average monthly PM_2.5_ (OR 1.1, 95% CI 0.97–1.3, *p* = 0.16).

**Conclusions:**

Consideration of only outdoor PM_2.5_ may obscure potentially modifiable risks for asthma symptoms. Specifically, this preliminary study suggests that cooking behaviors may contribute to the burden of PM_2.5_ in the homes of children with asthma and thus to asthma symptoms.

## Introduction

The Centers for Disease Control and Prevention reports that 24 million people in the US had asthma in 2015, including over six million children [1]. Pollution is an important risk factor for both development and exacerbation of asthma, with PM_2.5_ directly causing airway inflammation in mice models [2] as well as oxidative stress-induced autophagy *in vitro* [3]. Total outdoor particulate matter (PM) in a city’s air has been associated with the incidence of asthma in children, [4] and modeling of individual exposure to outdoor pollutants has shown significantly increased risk for the development of childhood asthma with increased exposure to PM [5]. Thus, pollution may be an important risk factor for, and contributor to, this very prevalent disease.

Indoor pollution may be particularly important to consider as people spend 80% or more of their time indoors [6]. In the developing world, cooking has been studied extensively as a source of indoor air pollution. Research in Guatemala has shown a higher prevalence of asthma among those living in homes where cooking was done over open fires as opposed to with improved, ventilated stoves [7]. However, the effects of PM_2.5_ from cooking on asthma symptoms in the developed world are underexplored.

In the developed world, indoor PM_2.5_ levels in New York were recently correlated with the risk of wheezing symptoms [8], and 70% of indoor PM_2.5_ originates from indoor sources [9], emphasizing the importance of exposures to PM_2.5_ from cooking, heating, house dust contaminated with mites and tobacco use when considering exposure in children with asthma. Exposure to nitrogen dioxide from cooking with natural gas has been associated with an increased risk of asthma exacerbations [10,11]. In the US National Health and Nutrition Examination Survey (NHANES) III (1988–1994), there were lower odds of asthma in households where parents reported using ventilation during use of their gas stoves [12]. A recently published report of measured PM_2.5_ in 13 residences in Seoul demonstrated differences depending on the type of ventilation used during a cooking task [13]. A few studies of cooking exposures in restaurants also have shown markedly elevated PM levels associated with particular types of cooking (especially frying)[14,15] and have even shown associations between airborne particles from cooking and inflammatory markers in exposed people[16,17]. In addition, recent work has demonstrated that some of the airborne particles produced from cooking can result from fuel combustion (gas) [18] as well as from organic material that is adsorbed to the cooking surfaces even in the absence of fuels which produce particles when combusted (such as electric stoves). [19]

The impact of tobacco smoke exposure on childhood asthma is well-known, and the presence of a smoker in the home greatly increases indoor PM_2.5_ levels[20]. Although the percentage of children in the US exposed to secondhand smoke has decreased in recent years [21,22], more than half of children with asthma in the US live with a smoker [23–25], with the highest prevalence (70%) among low-income families [25]. Thus, cigarette smoke exposure is also an important factor to consider.

The aim of this work was an assessment of the associations between household behaviors and home PM_2.5_ levels in an urban setting, as well as an exploration of the relationships between home PM_2.5_ levels and asthma severity.

## Materials and methods

**Fig 1:**
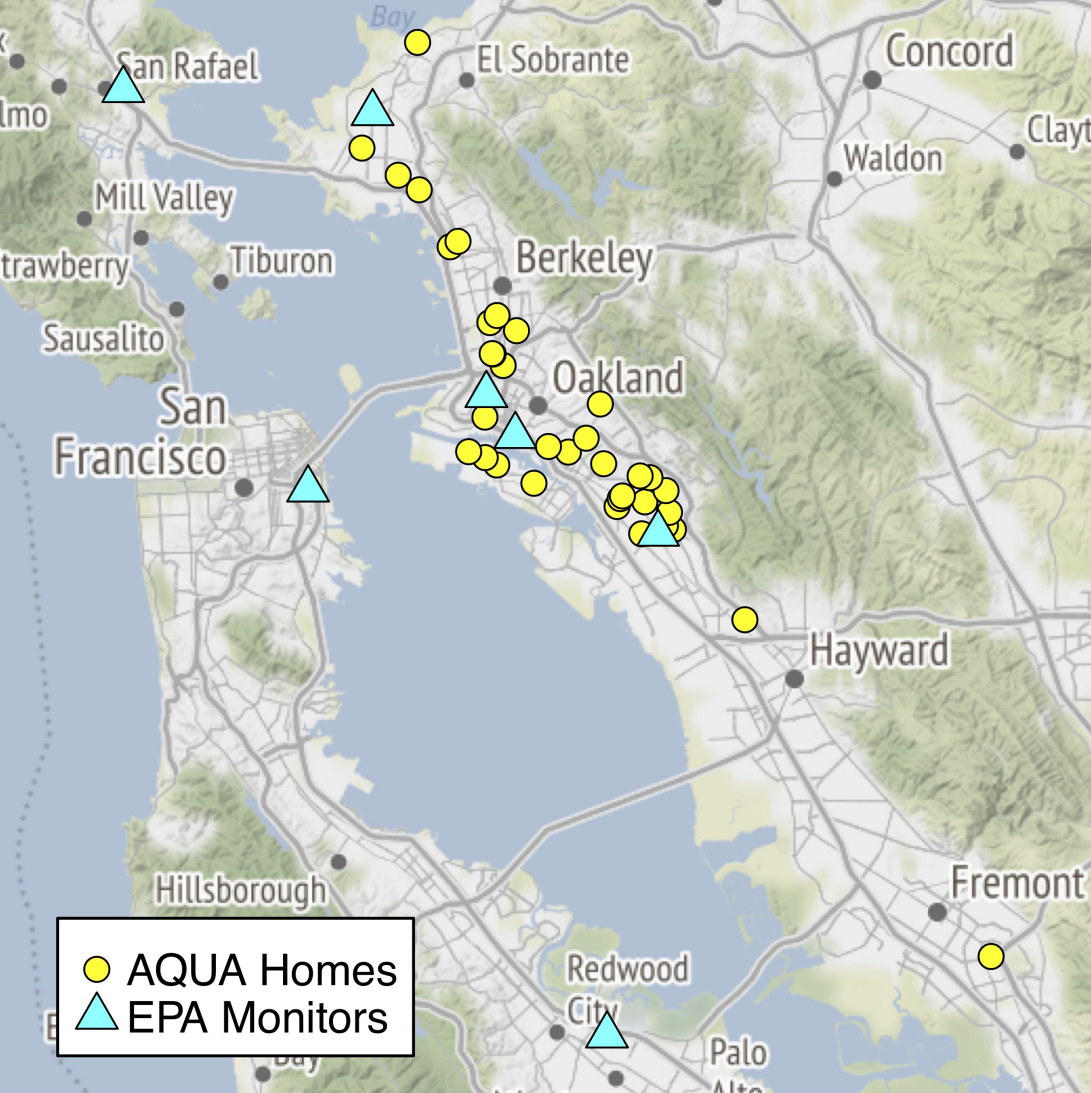
Map of the San Francisco Bay Area with locations of AQUA study households and local EPA monitoring stations noted. (Map tiles by Stamen Design, under CC BY 3.0. Data by OpenStreetMap under ODbL).

THE AQUA study (Tobacco in the Home Environment and Air Quality Undermining Asthma) was a dual cohort study of children with asthma (n = 35 total) in the San Francisco Bay Area, with one cohort exposed to tobacco smoke in the home (n = 16) and the other not exposed. The study was initially designed to determine whether an inexpensive optical PM_2.5_ sensor (the Portable University of Washington Particulate Matter Sensor, PUWP) could distinguish between homes with cigarette smoke exposure and those without. However, this report is focused on an analysis of the associations between household behaviors, home particulate levels and asthma severity. Locations of study homes and their nearest EPA monitors are visualized in Fig 1. Air quality (PM_2.5_) data were collected over a one-month period in each home. Study participants were run in three groups of 12–14 families in November 2015, February 2016 and April 2016.

The study was approved by the Committee on Human Research at UCSF Benioff Children’s Hospital of Oakland (IRB #2014–082), with agreement from the Committee for the Protection of Human Subjects at the University of California, Berkeley. Written informed consent was obtained from parents, adult participants and asthmatic children over the age of 7. Those under age 7 gave verbal assent.

### Participants

**Fig 2:**
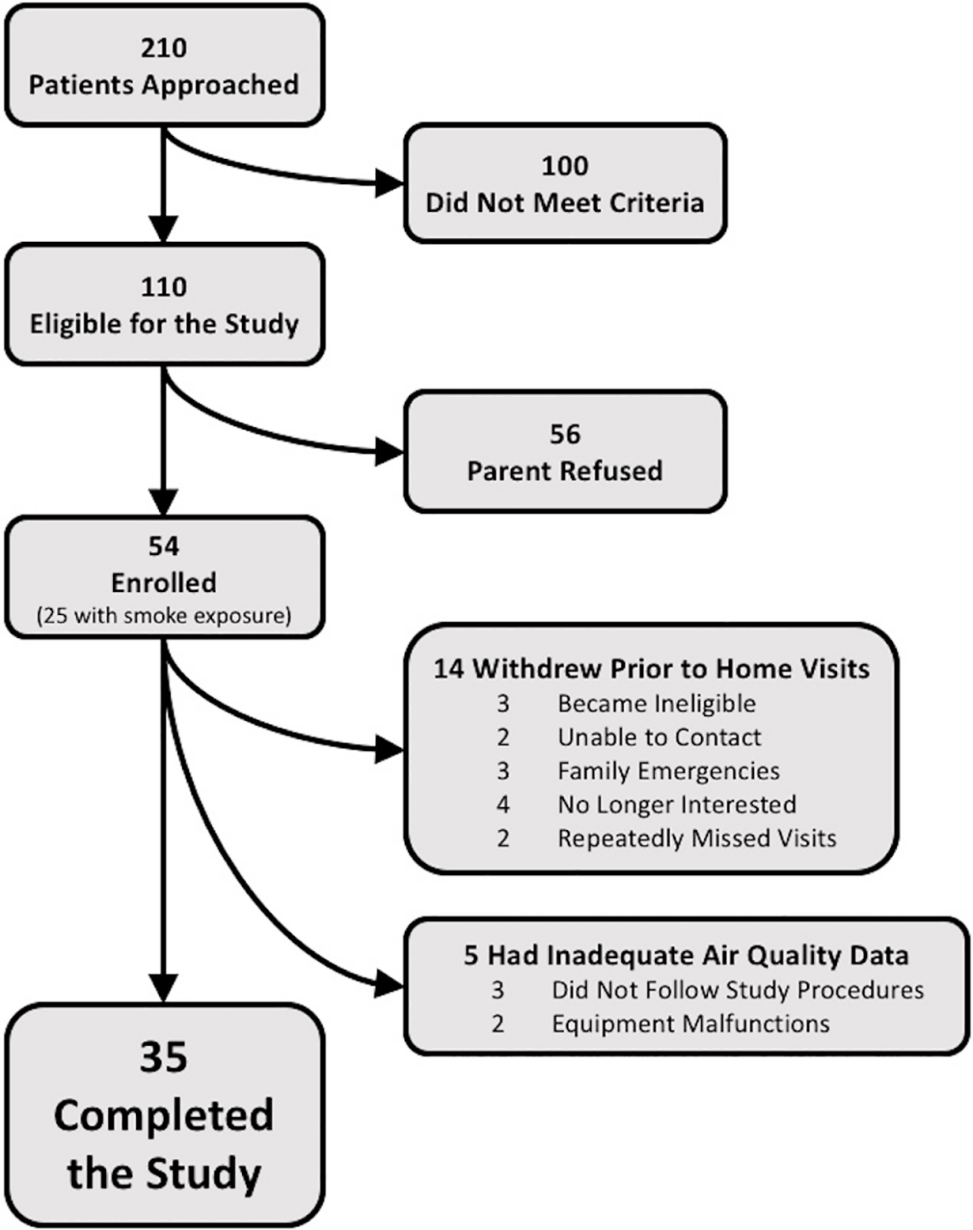
Summary of THE AQUA study enrollment. The participants who were noted to have become ineligible in the lower right box are one child diagnosed with sleep apnea and two whose housing situations became unstable after the time of initial enrollment but prior to the onset of study visits.

The participants were children 6–10 years old with physician-diagnosed asthma but no other major illnesses living in the East Bay Area of Northern California and attending primary care, asthma or pulmonary clinics at UCSF Benioff Children’s Hospital Oakland. Children were excluded if they had severe asthma exacerbation requiring systemic steroids in the last 3 months, were not compliant with asthma medications, were using albuterol daily and not on maximal controller therapy, a smoker in the home was a minor, were another participant’s sibling, or their parent or adult smoker was functionally illiterate in written English. Enrollment data are summarized in Fig 2.

### Home sensors

The PUWP sensor, a low-cost optical sensor which is sensitive to ambient fine PM approximating PM_2.5,_ [26] was placed in the central living area of each participant’s home. Central living area was defined as the area where the family (including the child with asthma) spent time, and was determined by the participant families. The PUWPs count particles from 0.75 to 6 μm in diameter, with a detection limit of 1 μg/m^3^, [26] which provides a good approximation for PM_2.5_. A commercially available optical PM monitoring device (Dylos DC1100 or DC1700, Riverside, CA, USA) was co-located in 29 of the 35 homes (limited by the number of devices available to us) as it has been successfully deployed in other studies.^,^ [27,28] The Dylos devices measure particles 0.5 μm and up, we used their small particle counts, which reflect particles of 0.5 to 2.5 μm. Air quality sensors were installed in a tamper-resistant container with a thermometer and humidity sensor (one of two similar models from Lascar electronics, either the EL-USB-2 or HT-10). An additional temperature/humidity sensor was placed in the kitchen, next to the stove. Five families of the 40 who completed study procedures had indoor air quality data excluded from final analyses due to improperly followed procedures for data collection (such as unplugging or moving the sensor box; 3 households) or PUWP malfunction (2 households), leading to the final sample of 35 families. PUWP particle counts were converted to PM_2.5_ mass using a chamber calibration with a Grimm Particle counter (Ainring, Germany) and filter sample. The PUWPs, the Grimm and a filter were co-located in a chamber that was filled with varying concentrations of cigarette smoke. For each sensor a linear model was created relating sensor sensitivity to Grimm particle counts; these were then converted to mass concentrations using the collected filters. PM_2.5_ mass measurements were subsequently scaled using the best fit line of PUWP measurements compared to PM_2.5_ weight determined from the eight homes in which ambient PM_2.5_ concentration was determined by collection on PTFE filter and gravimetric analysis, in order to account for the real-world mixture of PM found in the homes. Homes for PTFE filter collection were selected to be representative of the larger sample. Dylos data were used for verification of PUWP data but not analyses as the total quantity of data saved by the Dylos devices was markedly less as a function of how the Dylos device saves data, including the fact that the devices do not power back on after brief power interruptions. PM_2.5_ masses calculated from the measurements from the PUWP sensors (using filter data for calibration) were used for all analyses.

### Study measures

Standardized interview and chart review forms were developed for this study. Staff were trained to perform spirometry following American Thoracic Society/European Respiratory Society performance criteria [29], using a laptop spirometer in the homes.

From the scaled PUWP PM_2.5_ mass data, monthly and daily means, medians and percent of time above two relevant ambient PM_2.5_ thresholds were calculated. Although there is no US Environmental Protection Agency (EPA) standard for indoor PM_2.5_, we assessed the extent to which indoor levels exceeded current outdoor standards. The US EPA ambient air quality standards for PM_2.5_ thresholds were used as thresholds (12 μg/m^3^ annual average; 35 μg/m^3^ 24-hour average) [30]. Temperature time series data were downloaded from the kitchen temperature sensor.

Asthma severity was assessed from initial intake interview and chart review (both performed by a pediatrician), noting the asthma severity category [31]. Categories are standardly defined as intermittent, mild persistent, moderate persistent or severe persistent asthma. As asthma diagnosis was taken from clinical history and chart review, these were dichotomized to intermittent and persistent asthma in order to minimize the variation between different clinician assessments. Dichotomous variables from the visit interviews included: smoke exposure status, heater use, stove hood fan use (most of the stoves were gas and most hoods exhausted to the outdoors), presence of pets, highest household education (some college or not) and household annual income (>$25,000 or not). Smoke exposure, heater and fan use were included as these are known contributors to indoor PM. [9] Presence of pets and measures of SES were included as these are known asthma modifiers. [32,33] Though race/ethnicity is known to be related to asthma diagnosis, [32]and asthma phenotypes, this was not included as a factor in the analyses as nearly all our participants were African-American.

EPA monitoring data for the study period [34] were used to calculate an average outdoor PM_2.5_ daily mean value for each household from inverse distance-weighted-squared daily mean values for the four closest EPA monitoring sites to that household (see local monitoring stations in Fig 1). California Department of Transportation data [35] were used to calculate distance to nearest major roadway (in the state highway system) for each household.

### Sample size calculation

As our study was initially powered to detect PM_2.5_ differences between homes with and without smokers, prior data were used regarding PM_2.5_ levels in the homes of children with asthma (who live with or without smokers). In a previous study [36] 24-hour mean PM_2.5_ levels in homes with smokers were found to be 33 μg/m^3^ compared to a mean of 4 μg/m^3^ in non-smoking homes (we estimated a standard deviation of 33 from their reported data). With 20 families in each group, our study would have 0.79 power (with alpha 0.05) to reject the null hypothesis that PM_2.5_ levels in households with smokers were the same as those without. Due to difficulty enrolling participants and scheduling visits for families with smoke exposure our final sample with complete air quality data had only 13 families with smoke exposure, though we had data for a month rather than 24 hours as in the Semple et al. study. {Semple:2013hg}

Using data from a study which assessed PM_2.5_ levels during cooking with different ventilation patterns, [13] a post-hoc power analysis for the cooking ventilation-PM exposure can be performed. Median PM levels in the 2 hours after cooking were 1800 μg/m^3^ in homes that had windows open but did not use the range hood and 500 μg/m^3^ in homes that did use the range hood. With a sample the size of ours (29 homes which used the range hood, and 6 that did not we would have 0.83 power (with alpha 0.05) to detect a similar difference in the medians. However, as our households were not using their range hoods for all cooking episodes and because our data are for more than the 2 hours after each cooking interval, our power would be expected to be much lower.

A post-hoc power analysis given the enrollment we achieved yields a power of 0.71 (alpha 0.05) for detecting difference between homes with and without a smoker. Power was not calculated for the cooking ventilation analysis as no similar data could be located.

### Statistical models

Statistical analyses were performed using the statistical language R. [37] A spreadsheet of summarized and de-identified data are available in S1 Table. Monthly summary measures of PM_2.5_ exposure in the household were compared between households with and without smokers using Wilcoxon Rank-Sum tests (using wilcox.test from base R) because the data were markedly skewed. Exact p values and non-parametric confidence intervals were computed using the Hodges Lehman estimator, except for the regression predicting percent of time greater than 35, for which a normal approximation was required. [38] A permutation test was also run for each model using the permTS function from the perm package in R, [39] as a non-parametric test which makes fewer assumptions that the Wilcoxon (such as not requiring a normal approximation for confidence intervals and not assuming equal variance).

**Fig 3:**
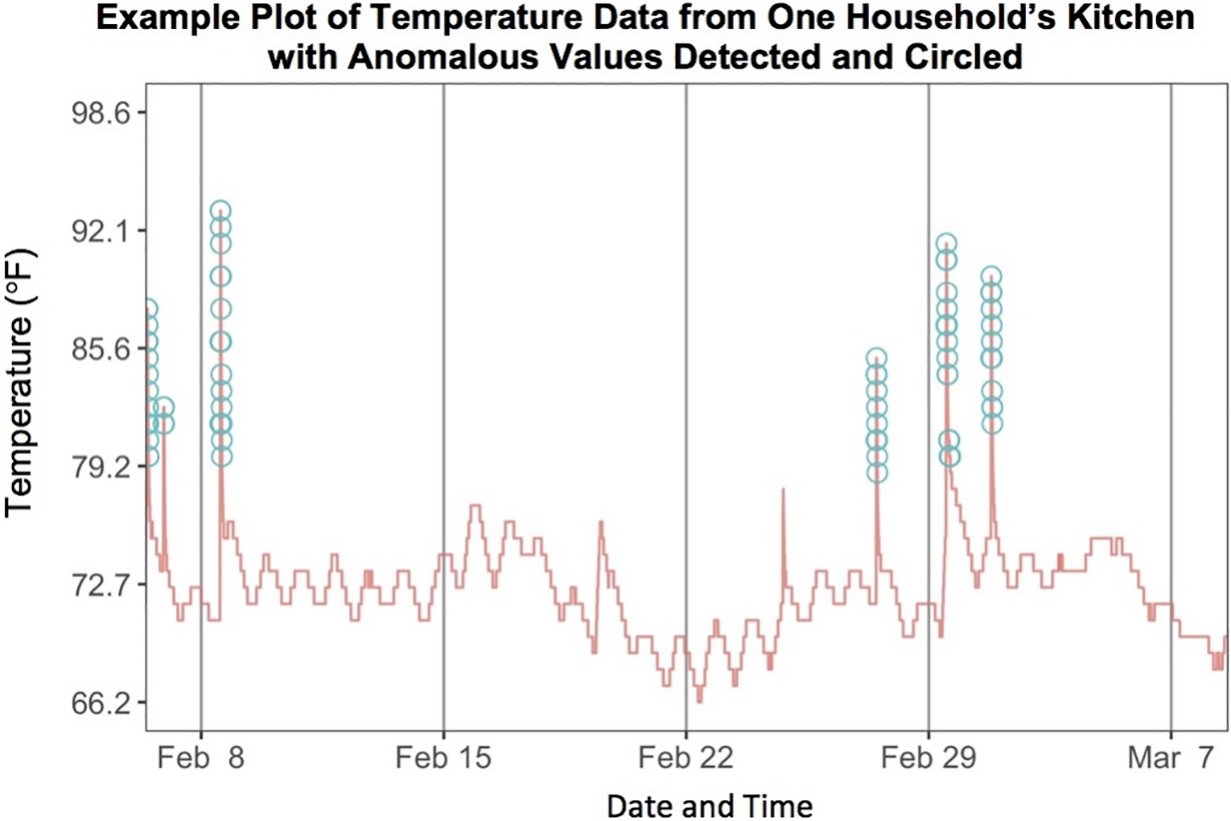
Example of kitchen temperature time series decomposition analysis results. This plot displays the recorded kitchen temperatures with anomalous increases in temperature (presumed cooking times) circled.

Anomalies in the kitchen temperature were determined using a package (‘anomalyDetection’ [40]) which performs statistical time series decomposition in R using a seasonal hybrid extreme studentized deviate (S-H-ESD) model with an alpha threshold of 0.05 (one-tailed). These anomalies were used to identify cooking intervals (Fig 3). For each household a median PM_2.5_ was calculated for both cooking and non-cooking intervals. These were then compared using a paired t-test (t.test in R). We excluded one extreme outlier which had significantly higher PM levels during cooking compared to any of the others, in order to be more conservative.

Linear regression models, using the lm function in R, were used to predict summary PM_2.5_ measures, using household behaviors and factors. Model assumptions were verified. Summary PM_2.5_ measures included mean (as this is used by regulatory bodies), as well as median and percent of time above the regulatory thresholds (as these are likely statistically more appropriate for skewed data.)

Asthma severity (intermittent versus persistent) was regressed with household factors and PM_2.5_ measures using logistic regression (the glm function in R). Household factors included presence of a smoker, presence of pets, distance to roadway, and income (dichotomized into above and below $25000 annually). Confidence intervals for the regression coefficients were calculated using profiled log likelihoods[41] and the Hosmer Lemeshow goodness of fit test was performed [42] with the Resource Selection package in R. [43]

## Results

Demographic data for the study population are summarized in Table 1, which are reflective of the clinic population from which the participants were recruited.

**Table 1:** Demographic data.

Characteristic	Total (n = 35)	Those that used the Stove Hood (n = 29)
**Child with Asthma**		
Sex		
*female*	19 (54%)	17 (59%)
Race/Ethnicity		
*Hispanic/Latino*	2 (6%)	2 (7%)
*White*	3 (9%)	2 (7%)
*Black*	28 (80%)	23 (79%)
*Asian*	1 (3%)	1 (3%)
*Other*	1 (3%)	1 (3%)
Asthma Severity		
*Mild Intermittent*	10 (29%)	9 (31%)
*Mild Persistent*	11 (31%)	10 (34%)
*Moderate Persistent*	13 (37%)	9 (31%)
*Severe Persistent*	1 (3%)	1 (3%)
Parent Reported Allergies		
*Yes*	14 (40%)	13 (45%)
Mean Age (years)	8.5 (1.6)	8.5 (1.6)
*Mean BMI percentile*	79^th^ (21)	80^th^ (22)
**Household**		
Single Family Home		
*Yes*	15 (43%)	12 (41%)
Number of household members	4.3 people (1.7)	4.2 (1.7)
Family Household Income		
*$25*,*000 or below*	22 (63%)	18 (62%)
*$25*,*001–50*,*000*	9 (26%)	8 (28%)
*$50*,*001–75*,*000*	1 (3%)	0 (0%)
*$75*,*001–100*,*000*	1 (3%)	1 (3%)
*$100*,*001 and above*	2 (6%)	2 (7%)
Highest Household Education Level		
*Did not graduate high school*	3 (9%)	3 (10%)
*High school graduate*	4 (11%)	4 (14%)
*Some college education*	14 (40%)	13 (45%)
*Has a college degree*	14 (40%)	9 (31%)
Family Reports using Stove Hood		
*Yes*	29 (83%)	29 (100%)
Household Member who smokes		
*Yes*	13 (37%)	10 (34%)

Data presented as number (%) or mean (sd).

### Household PM_2.5_ and contributing factors

Summaries of exposure data for the group that used their hood fan compared to those that did not are displayed in Table 2, with statistics for the comparison between these groups (without controlling for other factors). Note that the estimation of central tendency is the median of the differences between the samples, not the difference of the medians.

**Table 2:** Summaries of exposure measures by group.

Characteristic	Median PM_2.5_ (IQR)	Wilcoxon Estimated Difference (CI)	Wil-coxon p value	Permu-tation p value
Mean Monthly PM_2.5_ (μg/m^3^)		3.3 (-3.8–17.6)	>0.20	= 0.11
*Uses Hood Fan (n = 29)*	8.96 (6.57–11.70)			
*Does Not Use Hood Fan (n = 6)*	14.08 (5.44–25.42)			
Median Monthly PM_2.5_ (μg/m^3^)		2.1 (-3.0–7.6)	>0.20	= 0.09
*Uses Hood Fan*	6.67 (4.87–9.18)			
*Does Not Use Hood Fan*	8.55 (3.99–12.16)			
% of Hours the PM_2.5_ >12 μg/m^3^		5.6 (-3.8–46.4)	>0.20	= 0.06
*Uses Hood Fan*	7.81% (4.07–18.84)			
*Does Not Use Hood Fan*	28.71% (5.41–50.37)			
% of Hours the PM_2.5_ >35 μg/m^3^		2.3 (-0.44–18.0)	= 0.15	<0.01*
*Uses Hood Fan*	0.95% (0.37–2.15)			
*Does Not Use Hood Fan*	8.99% (0.96–18.09)			
Distance to Nearest Roadway (m)		-63 (-254-239)	>0.20	>0.20
*Uses Hood Fan*	266.3 (147.0–416.3)			
*Does Not Use Hood Fan*	130.9 (98.7–442.7)			
Outdoor PM_2.5_ Level (μg/m^3^)		0.23 (-1.4–1.8)	>0.20	>0.20
*Uses Hood Fan*	7.60 (6.99–8.69)			
*Does Not Use Hood Fan*	8.10 (7.07–9.43)			

Summaries and results of Wilcoxon rank sum tests comparing four measures of household PM_2.5_ exposure as well as other covariates between those households that used the fan above the stove and those that did not. Significant results (p <0.05) are indicated with an asterisk (*).

**Fig 4:**
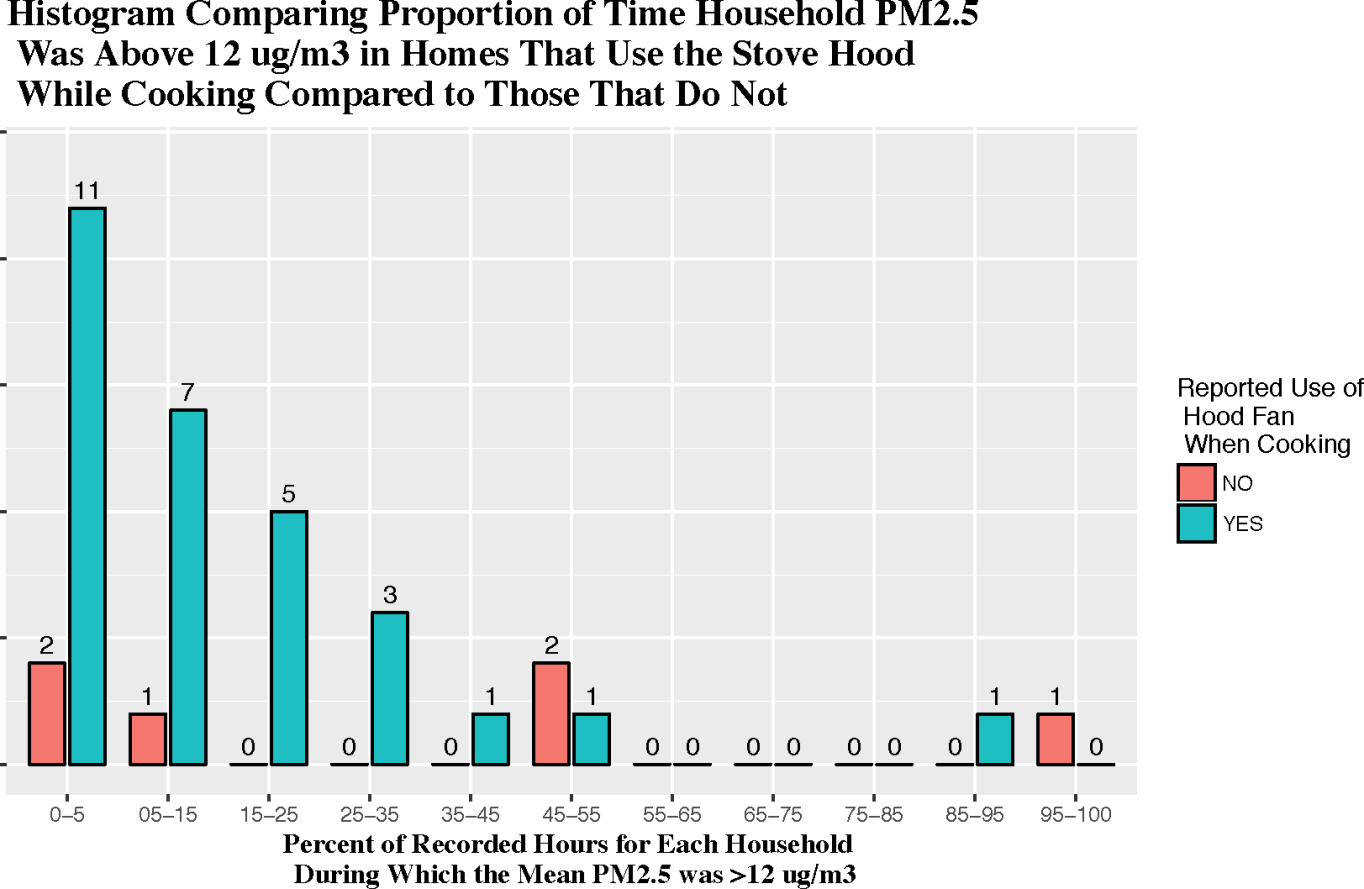
Comparison of particulate matter percent of hours above 12 μg/m^3^ between homes that reported no stove hood fan use and those that reported using it at least some of the time.

Linear regressions with monthly PM_2.5_ summary values as the outcome were performed to evaluate the role of each of these characteristics when controlling for the other factors: hood fan use, presence of a cigarette smoker, distance from the highway, presence of pets, heater use and outdoor PM_2.5_ levels (Table 3). These models were also compared to larger ones that included pets, heater use and outdoor PM_2.5_ level, and those additions did not show improvements in model fit. Presence of a cigarette smoker in the household was significantly related to all four PM_2.5_ summary statistics: mean, median, percent of time >12 μg/m^3^ and >35 μg/m^3^. For example, homes with a smoker had monthly mean PM_2.5_ levels 9.3 μg/m^3^ higher than those without (95% CI 4.5–14.2), adjusted for hood fan use and distance to highway. Not using a hood fan was also strongly correlated with percent of time with very high PM_2.5_ levels in the home (with those who never used the hood fan spending 8.3% more time with levels >35 μg/m^3^ than those that used the hood fan at least some time, 95% CI 2.9–13.6 adjusting for smoke exposure and distance to the highway) (Fig 4). Percentages of time spent above the WHO indoor/outdoor ambient air quality guideline values (10 and 25 μg/m^3^)[44] were comparable to those using the EPA thresholds. There was a trend toward significance in the differences between those who used the hood fan and those that did not in mean, median and percent of time at moderately high PM_2.5_ levels. Notably the effect sizes for use of a hood fan were similar to the effect sizes for smoke exposure. Of the 35 homes studied, 26 had hood fans that vented to the outdoors, 6 had vents which re-circulated air into the kitchen and 3 had no stove vent at all. Distance to the nearest roadway was unexpectedly related to an increase in household PM, however, this effect was not significant.

**Table 3:** Model fit statistics and coefficients from linear regressions.

	Mean PM_2.5_	Median PM_2.5_	% Time >12 μg/m^3^	% Time >35 μg/m^3^
Model Adjusted R^2^	0.3355	0.1249	0.1456	0.396
Residual Standard Error	6.7	4.8	22.4	5.8
Smoke Exposed	9.3	3.6	14.7	7.3
	(4.5, 14.2)	(0.1, 7.0)	(-1.4, 30.8)	(3.1, 11.4)
	*p< 0*.001*	*p<0*.05*	*p* = 0.07	*p<0*.005*
Hood Fan Use	-4.6	-3.4	-18.7	-8.3
	(-10.8 1.6)	(-7.9, 1.0)	(-39.3, 1.98)	(-13.6, -2.9)
	*p = 0*.*14*	*p = 0*.*13*	*p* = 0.07	*p<0*.005*
Distance to Highway (reported per km)	4.9	1.5	18.1	0.1
	(-4.4, 14.4)	(-5.2, 8.3)	(-13.4, 49.6)	(-8.1, 8.3)
	*p>0*.*20*	*p>0*.*20*	*p>0*.*20*	*p>0*.*20*

Model Fit Statistics and Coefficients from Linear Regressions with each of the four monthly summary PM_2.5_ statistics as the outcome variable, with confidence intervals in parentheses and p values below. Significant results (p <0.05) are indicated with an asterisk (*).

To further explore the relationship between PM_2.5_ levels and cooking in this population, median PM levels during cooking and non-cooking intervals were compared in a pairwise fashion (within household). Median PM_2.5_ during cooking intervals was, on average, 0.88 μg/m^3^ higher than levels when cooking was not done (*p* <0.005, 95% CI 0.33–1.42).

### Clinical asthma outcomes and PM_2.5_

The relationship between mean PM_2.5_ and asthma severity approached significance (*p* = 0.16, OR 1.6, 95% CI 0.88–3.7) for persistent versus intermittent asthma for every 5 μg/m^3^ increase in the monthly mean PM_2.5_ level, adjusted for cigarette smoke exposure, household income, presence of pets, and distance to highway. Asthma severity was not significantly associated with percentage of time above the regulatory cut points.

Because cigarette smoke exposure is related to PM_2.5_ levels, a post hoc analysis of the relationship between asthma severity and PM_2.5_ levels in the participants with no cigarette smoke exposure in the household was performed (n = 22). In this small sample, there was an odds ratio of 10.2 (CI 0.91–946.5, p = 0.17) for persistent versus intermittent asthma for every 5 μg/m^3^ increase in the monthly mean PM_2.5_, adjusted for socioeconomic status, presence of pets and distance to highway.

## Discussion

In this study of the home air pollution experienced by children with asthma there were strong associations with both the presence of a smoker in the home and use of ventilation during cooking and household particulate matter (PM_2.5_) levels. Moreover, homes had higher PM_2.5_ during cooking intervals. Finally, there is a trend toward increased odds of persistent asthma with increased PM_2.5_ levels. The findings that during times of cooking (over mostly gas stoves, though some electric) there were higher PM_2.5_ levels and that use of a hood while cooking was associated with less time spent with high PM_2.5_ levels (>35μg/m^3^) are particularly notable. While there are many potential contributors to indoor air pollution, cooking exposures are not often recognized as a risk factor for asthma exacerbations in the developed world and could be a potential target for interventions.

We found a trend between monthly mean indoor PM_2.5_ levels and asthma severity, even in children with asthma who lived in homes without smoke exposure (whose total exposure was lower). This may be a relevant relationship that requires a larger sample size to explore.

It is well-documented that outdoor air pollution is a risk factor for development and exacerbations of asthma [45,46]. Indoor pollution is of increasing concern as people spend more time indoors [6]. Indoor household air pollution can result from many sources, but much of the indoor pollution burden can be related to cooking [9,10], although homes with smokers have markedly higher PM_2.5_ levels [27]. Development of asthma is more likely in children who live with smokers, and this relationship is likely at least partially mediated through PM_2.5_ exposure [47]. This provides additional motivation for smoking cessation in families with children who have asthma.

Regarding cooking exposures, an analysis of NHANES data has shown that children whose parents report using ventilation while cooking with gas stoves are less likely to be diagnosed with asthma than children whose parents do not ventilate their gas stoves. {Kile:2014fd} Certain types of cooking, particularly frying, [15] are known to create large amounts of particulate matter. Thus, encouraging minimal use of frying could have respiratory benefit as well as dietary benefits for the household. Cooking events also include exposure to NO_2_, ultra-fine particulate matter and polycyclic aromatic hydrocarbons, which can also be pro-inflammatory. [17,18] This study also adds evidence that use of ventilation during cooking has measurable effects on PM_2.5_ in the homes children with asthma in urban settings and suggests that indoor home PM_2.5_ levels may be related to diagnosed asthma severity.

Of note, our study was performed in a study population that was primarily low-income, living in urban areas and of non-Hispanic black race. Both socioeconomic status and measures of neighborhood disorder are known risk factors for asthma, [32] so these children have many risk factors for asthma in their milieu. Moreover, African-American children have the highest prevalence of asthma among racial groups in the U.S., with some unique genetic [48] and inflammatory patterns (increased eosinophilic airways inflammation). [49] These sorts of behavioral interventions for household air quality may therefore be particularly important for this population, which is high risk for asthma morbidity.

Because we collected continuous monitoring data, we obtained a more complete picture of home PM_2.5_ exposures than is usually available. This allows for a more in-depth exploration of the contributions of indoor sources to a child’s total PM_2.5_ exposures. The lack of indoor air quality guidelines in the United States led us to use outdoor standards as a best proxy. Although we found significant associations with cooking and smoking behaviors, a home’s PM_2.5_ levels cannot be predicted based on these characteristics alone due to the large amount (at least 60%) of unaccounted for variation in our exposure models. We likely underestimated the magnitude of the associations between exposure to PM_2.5_ and asthma outcomes, as our continuous PM_2.5_ monitoring data in homes included times that the children were away from home, although results were similar when restricting to the hours that the child usually spent at home. While we observed a trend between small differences in monthly household PM_2.5_ and persistent asthma, our relatively small sample size limits our ability to interpret the relationships of PM_2.5_ exposure with clinical measures of asthma.

## Conclusions

Household behaviors can increase the exposure to PM_2.5_ that children with asthma experience in their own homes. As expected, the presence of a cigarette smoker in the home was a significant contributor to the household PM_2.5_ burden. In this urban, low-income population, use of the hood fan over the stove was also a significant factor in the number of airborne particles to which the children were exposed. Interestingly, the percent of time spent at high PM_2.5_ levels is more closely related to hood fan use than to smoking status in our sample. While these relationships should be further explored in detail, it may be worthwhile for physicians counseling children with asthma and their parents to consider discussion of ventilation during cooking as a way of minimizing particulate matter exposure, as there is little associated risk.

## Supporting information

S1 Table Deidentified dataset. (CSV)
